# *In vitro* study on anti-*Helicobacter pylori* effects of DL-3-n-butylphthalide-loaded silk fibroin nanoparticles

**DOI:** 10.3389/fmicb.2025.1750216

**Published:** 2026-01-12

**Authors:** Jie Cui, Meiyun Chen, Haonan Li, Tianyi Zhang, Fengli Lin, Xiaoyan Shi, Junwei Jia, Chun Wang, Ruixia Wei, Guimin Zhang, Meicun Yao, Zhong Feng

**Affiliations:** 1 School of Pharmacy, Shandong University of Traditional Chinese Medicine, Jinan, China; 2 School of Medicine, Southern University of Science and Technology, Shenzhen, China; 3 School of Pharmaceutical Sciences (Shenzhen), Sun Yat-sen University, Shenzhen, China; 4 Graduate School, Tianjin University of Traditional Chinese Medicine, Tianjin, China; 5 State Key Laboratory of Integration and Innovation of Classic Formula and Modern Chinese Medicine, Lunan Pharmaceutical Group Co., Ltd., Linyi, China

**Keywords:** antibacterial mechanism, Dl-3-n-butylphthalide, *Helicobacter pylori*, metabolomics, silk fibroin nanoparticles

## Abstract

*Helicobacter pylori* (*H. pylori*) primarily colonizes the gastric mucosal epithelium in humans and is considered the strongest risk factor for gastric cancer. Current clinical eradication regimens rely on proton pump inhibitor (PPI)-based triple or quadruple antibiotic therapies. However, rising antibiotic resistance and reinfection rates greatly compromise their efficacy, underscoring the need for effective alternative treatments. This study identified potent *in vitro* anti-*H. pylori* activity of DL-3-n-Butylphthalide (NBP). Using silk fibroin as a carrier, a nanoparticle delivery system was constructed to further investigate the antibacterial potential and mechanism of NBP. Uniform and stable nanoparticles were successfully prepared, exhibiting an encapsulation efficiency of 45.80 ± 1.5% and drug loading of 15.27 ± 0.8%. Subsequently, Fourier transform infrared (FTIR) spectroscopy and X-ray diffraction (XRD) were performed, and the results collectively confirmed the successful encapsulation of NBP. The minimum inhibitory concentrations (MICs) of NBP against standard strains and clinical isolates of *H. pylori* ranged from 4 to 16 μg/mL, and the minimum bactericidal concentrations (MBCs) were between 8 and 32 μg/mL. The nanoparticles exhibited enhanced antibacterial and bactericidal activities, with MICs ranging from 2.5–10 μg/mL and MBCs from 5 to 20 μg/mL. When used in combination with four antibiotics, the interaction showed additive, irrelevant effects with no antagonistic phenomenon. Both NBP and its nanoparticles downregulated the expression of cytotoxin-related genes, vacuolating toxin (cagA, vacA), flagellum genes (flaA, flaB), and urease genes (ureA-B, ureE-H, nixA) mRNA, inhibited *H. pylori* motility and urease activity, destroyed the bacterial structure, and significantly reduced the expression of relevant virulence proteins. Integrated untargeted metabolomics and network pharmacology analysis further revealed five key metabolic pathways and seven core targets underlying the anti-*H. pylori* action of NBP. These findings highlight the promising role of NBP, particularly in a nanoformulation, as a potential multi-mechanistic therapeutic agent against *H. pylori* infection.

## Graphical abstract

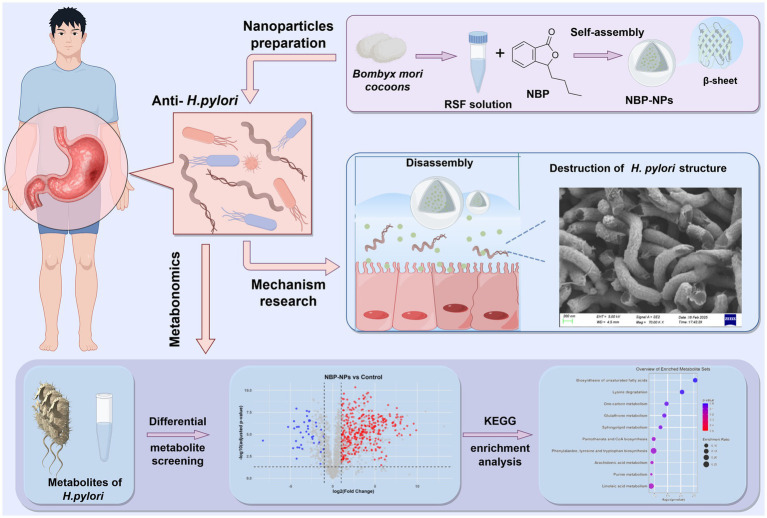

## 1 Introduction

*Helicobacter pylori* (*H. pylori*) is characterized as a spiral-shaped, Gram-negative bacterium that thrives in microaerophilic conditions. This bacterium possesses flagella and a chemotaxis system that allows it to develop various strategies to adapt to the harsh environment of the gastric corpus and use chemotactic motility to migrate to the gastric antrum and establish a persistent infection (Lertsethtakarn et al., 2011). *H. pylori* infection is closely related to chronic gastritis, peptic ulcers, and gastric adenocarcinoma, making it the strongest known risk factor for gastric cancer (Amieva and Peek, 2016; Zavros and Merchant, 2022). Epidemiological statistics show that approximately half of the world’s population is infected with *H. pylori*, with infection rates varying significantly between different regions and countries. Higher infection rates are found in Africa (79.1%) and Latin America and the Caribbean (63.4%), while lower rates are seen in Oceania (24.4%) and Switzerland (18.9%) (Burucoa and Axon, 2017; Hooi et al., 2017).

In the 1990s, a proton pump inhibitor (PPI) combined with one or two antibiotics achieved a 90% eradication rate of *H. pylori* (European Helicobacter pylori Study Group, 1997). However, the global misuse of antibiotics has led to increased resistance, with *H. pylori* evading antibiotic activity through mutations in cell targets, changes in efflux systems or cell membrane permeability, or the secretion of *β*-lactamases and various virulence factors (Gong and Yuan, 2018). First-line treatments based on individual antibiotic sensitivity are effective but challenging to implement (Fallone et al., 2019). In China, *H. pylori* exhibits high resistance to clarithromycin, levofloxacin, and metronidazole, leading to eradication efficiency of less than 80% with traditional triple therapy. Consequently, bismuth-based quadruple therapy has been included in clinical guidelines as the first-line treatment for *H. pylori* in China (Hu et al., 2017). Despite considerable efforts in recent years to introduce novel treatments for *H. pylori*, such as probiotics, plant extracts, or biofilm formation inhibitors, over 20% of patients still experience treatment failure (Roszczenko-Jasińska et al., 2020). Therefore, there is an urgent need to find new agents with high selectivity against *H. pylori*.

DL-3-n-Butylphthalide (NBP), a colorless oily liquid, is the first national Class I new drug with independent intellectual property rights in China for cerebrovascular treatment and has been approved by the FDA for Phase II clinical trials for ischemic stroke. NBP was initially extracted from the seeds of *Apium graveolens* (Chinese celery) and exhibits multiple pharmacological activities, including microcirculation reconstruction, mitochondrial function protection, oxidative stress inhibition, and neuronal apoptosis suppression (Que et al., 2021; Zhao et al., 2014; Zhao et al., 2013), as well as antiplatelet, antithrombotic, and endothelial progenitor cell mobilization effects. It holds great promise in the treatment of neurological diseases and cerebrovascular disorders (Chen X. Q. et al., 2019; Peng et al., 2004). However, studies have demonstrated that NBP’s therapeutic potential extends beyond these areas, including in metabolic diseases, neuroinflammation, and immunity (Xu et al., 2021; Zhang Y. et al., 2022). These cross-disciplinary effects suggest that NBP may be a pleiotropic molecule, but its specific targets and translational applications still require further exploration. To date, NBP has not been explored in gastrointestinal diseases. This study discovered its potent inhibitory effect on *H. pylori* and investigated its potential anti-*H. pylori* mechanism through *in vitro* experiments.

NBP exhibits a logP value of 3.08, indicating high lipophilicity, limited solubility and absorption in the gastrointestinal environment, and low oral bioavailability. Additionally, highly lipophilic drugs tend to accumulate in the gastric mucosa, potentially causing local irritation. Silk fibroin (SF) is a natural protein polymer widely present in *Bombyx mori* cocoons, comprising over 70% of the total cocoon weight. It offers advantages such as low immunogenicity, biocompatibility, and ease of large-scale production. SF has broad development prospects in tissue engineering, wound dressings, and drug delivery systems (Kasoju and Bora, 2012; Mazurek et al., 2022; Wani et al., 2020; Zhou et al., 2022). Furthermore, SF exhibits excellent tunable properties, especially self-assembly capabilities, enabling controlled transitions between various aggregation forms through artificial intervention (Bai et al., 2013). Its porous scaffold can be degraded by macrophages, achieving controlled biodegradation (Wang et al., 2008). This study used SF as a carrier to construct a nanoparticle delivery system, explored the *in vitro* anti-*H. pylori* activity of NBP, and investigated its possible mechanisms.

This study employed non-targeted metabolomics to characterize the metabolic perturbations in *H. pylori*, integrated with network pharmacology and molecular docking to predict potential therapeutic targets, thereby providing an in-depth investigation into the antibacterial mechanism of NBP. Molecular docking is a widely applied computational approach used to predict the interaction patterns and binding affinities between active compounds and target proteins (Qanash et al., 2023). It provides valuable insights into the possible mechanisms of action by simulating how small molecules fit within the active sites of microbial or cellular enzymes. Docking analysis helps identify key binding residues, estimate interaction energies, and characterize the stability of ligand–protein complexes (Yahya et al., 2022; Alsalamah et al., 2023). By integrating in silico predictions with *in vitro* biological assays, molecular docking strengthens the understanding of structure–activity relationships and supports the rational design of more effective antimicrobial and anti-inflammatory agents (Al-Rajhi et al., 2023). In studies evaluating active compounds, docking serves as a complementary tool that highlights the therapeutic potential of these compounds and guides further experimental validation.

## 2 Materials and methods

### 2.1 Chemicals and reagents

Butylphthalide was obtained from Shandong New Era Pharmaceutical Co., Ltd. (Lot#: 694220802). Sterile defibrinated sheep blood was obtained from Hongquan Biotechnology (Guangzhou, Guangdong, China). Brain Heart Infusion (BHI) and Columbia agar were purchased from Oxoid Ltd. (Basingstoke, Hants, United Kingdom). Phosphate-buffered saline (PBS), penicillin–streptomycin, and fetal bovine serum (FBS) were purchased from Gibco-Life Technologies LLC (Rockville, MD, United States). Clarithromycin (CLR) and metronidazole (MET) were obtained from Macklin Biochemical Co., Ltd. (Shanghai, China). Levofloxacin (LEF) was purchased from Target Molecule Corp. (Boston, MA, United States). Amoxicillin (AMO) was obtained from the National Institute for Food and Drug Control (Beijing, China). Urea and acetohydroxamic acid were purchased from Macklin Biochemical Co., Ltd. (Shanghai, China). Tween-20 was sourced from Biotopping (Beijing, China). Phenol red was purchased from Sigma. Beyotime Biotechnology (Shanghai, China) provided RIPA lysis buffer, PMSF, phosphatase inhibitor cocktail (50x), SDS-PAGE gel rapid preparation kits, BeyoColor™ prestained protein markers, and BeyoECL Star ultra-sensitive chemiluminescence detection reagent kits. Additionally, protease inhibitor cocktail was obtained from Roche (Basel, Switzerland), and anti-*H. pylori* CagA antibody was sourced from Santa Cruz Biotechnology (Dallas, TX, United States).

### 2.2 Silk fibroin extraction

Silk fibroin (SF) was extracted from *Bombyx mori* cocoons using an alkaline degumming method. Cocoons were cut into uniform fragments and boiled in a 0.5% (w/v) Na₂CO₃ solution. After boiling, the silk was rinsed three times with purified water. This degumming process was repeated three times, followed by overnight drying to obtain degummed silk fibers. The fibers were then dissolved in 9.5 M LiBr solution by heating in a 60 °C water bath (Phillips et al., 2004; Rockwood et al., 2011). Once fully dissolved, the solution was transferred into dialysis tubing (MWCO 3500 Da) and dialyzed against purified water for 3 days. The resulting regenerated silk fibroin (RSF) solution was centrifuged at 12,000 rpm for 10 min to remove insoluble impurities. The final RSF concentration was determined by the dry weight method.

### 2.3 Nanoparticles preparation

NBP-loaded nanoparticles (NBP-NPs) were prepared using a desolvation method (Perteghella et al., 2017; Seib et al., 2013). Under magnetic stirring at 600 rpm, a 10 mg/mL RSF solution was added dropwise to acetone. The mixture was then sonicated in an ice bath at 50% amplitude for 1 min. Subsequently, it was stirred in a fume hood for 1 h. The resulting nanoparticles (named SF-NPs) were collected by centrifugation at 12,000 rpm for 20 min, washed three times with purified water, freeze-dried, and stored at −20 °C for further use. The NBP-loaded nanoparticles (NBP-NPs) were prepared following the same procedure, beginning with the dissolution of 5 mg of NBP in acetone. Subsequently, 1 mL of the RSF solution (10 mg/mL) was added dropwise to the acetone solution under magnetic stirring. The subsequent steps of sonication, stirring, centrifugation, washing, and freeze-drying were performed as described above.

### 2.4 Characterization of nanoparticles

#### 2.4.1 Particle size, PDI and zeta potential

The particle size, polydispersity index (PDI), and zeta potential of the SF-NPs and NBP-NPs were measured using a Zetasizer Nano ZS (United Kingdom). Each sample was measured three times, with a 120-s equilibration for each measurement. Prior to analysis, samples were adequately diluted with purified water, and the measurement temperature was set at 25 °C.

#### 2.4.2 Encapsulation efficiency and drug loading evaluation

The encapsulation efficiency (EE) and drug loading (DL) of the NBP-NPs were analyzed using a high-performance liquid chromatography (HPLC) system (Thermo, Ultimate 3,000) with a Phenomenex Kinetex XB-C18 column (4.6 mm × 250 mm, 5 μm). The analysis conditions included UV detection at 228 nm, a column temperature of 25 °C, an injection volume of 10 μL, a flow rate of 1.0 mL/min, and isocratic elution with a mobile phase consisting of acetonitrile–water–acetic acid (55,45,1.8, v/v). Each measurement was performed in triplicate. The EE and DL were calculated using the following formulas:


$$\text{Encapsulation efficiency}(\%)=\frac{\text{Drug entrapped}}{\text{Initial drug added to prepare}\;\mathrm{NPs}}\times 100$$

$$\text{Loading capacity}(\%)=\frac{\text{Drug entrapped}}{\text{Weight of}\;\mathrm{NPs}\;}\times 100$$

Drug-loaded nanoparticles were dissolved in methanol and sonicated for 30 min. After centrifugation at 12,000 rpm for 5 min, the supernatant was collected for HPLC analysis.

#### 2.4.3 Morphological evaluation by scanning electron microscopy

The surface morphology of the nanoparticles was examined using a Sigma 500 field-emission SEM (ZEISS, Germany). A small amount of freeze-dried sample was spread evenly on a conductive adhesive stub, sputter-coated with gold under vacuum, and observed at appropriate magnifications under an inert atmosphere.

#### 2.4.4 Fourier transform infrared spectroscopy

FT-IR spectra of SF-NPs and NBP-NPs were acquired on a Nicolet iS50 FT-IR spectrometer (Thermo Fisher, United States). Freeze-dried powders of SF-NPs and NBP-NPs were mixed with potassium bromide (KBr) powder at a ratio of 1: 200 (w/w), ground thoroughly, and pressed into pellets. Spectra were recorded in transmission mode over the 400–4,000 cm^−1^ range. For pristine NBP, the determination was carried out by the smear method.

#### 2.4.5 X-ray diffraction

The crystallinity of pristine NBP, SF-NPs and NBP-NPs was assessed using an Empyrean X-ray diffractometer (PANalytical, Netherlands). Samples were placed on the sample holder. Cu–Kα radiation was used at 40 kV and 40 mA. Data were collected over a 2θ range of 3°–50°, with a step size of 0.02° and a scan speed of 0.1 s per step, all at room temperature.

### 2.5 *In vitro* anti-*Helicobacter pylori* activity of NBP and its nanoparticles

#### 2.5.1 *Helicobacter pylori* strains and culture conditions

Standard strains ATCC 700392 and ATCC 43504 were obtained from the American Type Culture Collection (ATCC, Manassas, VA, United States). Sydney strains SS1 and CSO1 were kindly provided by Shanghai University of Technology. Clinical isolates QYZ-001, QYZ-003, and QYZ-004 were generously gifted by Qingyuan Traditional Chinese Medicine Hospital (Guangzhou, China). All strains were confirmed by morphology, Gram staining, and biochemical tests. Antibiotic resistance breakpoints followed European Committee on Antimicrobial Susceptibility Testing (EUCAST 2019) guidelines. Strains were cultured on Columbia blood agar supplemented with 5% defibrinated sheep blood in a tri-gas incubator (37 °C, 10% CO₂, 5% O₂, 85% N₂). For assays, bacteria were inoculated into brain heart infusion (BHI) broth supplemented with 10% FBS (v/v) and shaken at 150 rpm, 37 °C (Chen et al., 2024).

#### 2.5.2 Minimum inhibitory concentration and minimum bactericidal concentration

MICs were determined by the microdilution broth method (Naccache et al., 2019). Bacterial suspensions (1 × 10^6^ CFU/mL) and serial dilutions of drug solutions were mixed 1:1 in 96-well plates and incubated at 150 rpm, 37 °C for 3 days. The MIC was defined as the lowest concentration showing a marked reduction in turbidity. BHI + 10% FBS served as the negative control; clarithromycin was the positive control; untreated bacteria were the growth control. Wells at MIC, 2 × MIC, and 4 × MIC were plated on Columbia blood agar and incubated for 3 days. The MBC was defined as the lowest concentration reducing viability by ≥99.9% compared to untreated controls. All experiments were performed in triplicate.

#### 2.5.3 Inhibiting kinetics assay

The standard strain ATCC 700392 and the clinical strain SS1 were exposed to NBP and NBP-NPs solutions at concentrations of 2 × MIC, 1 × MIC, and 0.5 × MIC under microaerophilic conditions at 37 °C with shaking at 150 rpm for 3 days. At time points of 0 h, 8 h, 12 h, 24 h, 28 h, 32 h, 36 h, 48 h, 60 h, and 72 h, 100 μL of bacterial suspension was sampled and the optical density at 600 nm (OD₆₀₀) was measured using a POLARstar Omega microplate reader (BMG Labtech). The negative control consisted of BHI medium supplemented with 10% FBS only, while the positive control consisted of clarithromycin at its MIC concentration.

#### 2.5.4 Fractional inhibitory concentration index

A checkerboard microdilution assay was used to evaluate the *in vitro* synergistic activity of NBP and NBP-NPs in combination with four antibiotics against *H. pylori* (Dutt et al., 2023). Serial two-fold dilutions of NBP, NBP-NPs, and the antibiotics were prepared (final concentrations ranging from 1/8 × MIC to 2 × MIC). Bacterial suspensions (1 × 10^6^ CFU/mL), antibiotic solutions, and NBP or NBP-NPs solutions were mixed in a 96-well plate at a ratio of 2:1:1 and incubated under microaerophilic conditions for 3 days. The Fractional Inhibitory Concentration Index (FICI) was calculated to evaluate the interaction between NBP and the antibiotic using the formula: FICI = (MIC of NBP in combination/MIC of NBP alone) + (MIC of antibiotic in combination/MIC of antibiotic alone). Here, MIC represents the minimum inhibitory concentration for each agent when used alone or in combination. The FICI values were interpreted as follows: FICI ≤ 0.5 indicated a synergistic effect, 0.5 < FICI ≤ 1.0 represented an additive effect, 1.0 < FICI ≤ 2.0 suggested an irrelevant effect, and FICI > 2.0 reflected an antagonistic effect. To ensure reliability, all experiments were performed in triplicate, and results were averaged for final analysis.

#### 2.5.5 RT-qPCR analysis of virulence gene expression

RT-qPCR was employed to assess the impact of NBP and its nanoparticles on the expression of *H. pylori* virulence genes (Lu et al., 2021). Mature *H. pylori* cultures were adjusted to a turbidity of 1.0 McFarland and incubated for 24 h. A 1 mL aliquot of bacterial suspension was diluted 1:50 in BHI broth supplemented with 10% FBS to establish the growth control group. For the treatment groups, NBP and NBP-NPs were added at MIC concentrations. All cultures were incubated at 37 °C under microaerophilic conditions with shaking for 12 h. Total RNA was extracted using the RNeasy Mini Kit (Qiagen, Germany). After quantification and normalization, reverse transcription was performed using the PrimeScript RT Master Mix (Takara, Japan). RT-qPCR was conducted on an Applied Biosystems 7,500 Fast Real-Time PCR System (Thermo Fisher Scientific, United States) using the SYBR Premix Ex Taq™ kit (Takara). Relative gene expression was analyzed using the 2^−ΔΔCt^ method, with the *16S rRNA* gene used as the internal reference. Changes in target gene expression were determined by comparing treated samples to the untreated control group. The specific primers used for amplification are listed in Table 1.

**Table 1:** The specific primers of *H. pylori* virulence genes.

Gene name	Sequences forward(F)	Sequences reverse(R)
*16S rRNA*	CCGCCTACGCGCTCTTTAC	CTAACGAATAAGCACCGGCTAAC
*cagA*	ATAATGCTAAATTAGACAACTTGAGCGA	TTAGAATAATCAACAAACATCACGCCAT
*vacA*	CTGGAGCCGGGAGGAAAG	GGCGCCATCATAAAGAGAAATTT
*flgA*	ATTGGCGTGTTAGCAGAAGTGA	TGACTGGACCGCCACATC
*flgB*	ACATCATTGTGAGCGGTGTGA	GCCCCTAACCGCTCTCAAAT
*ureA*	ACCGGTTCAAATCGGCTCAC	CAAACCTTACCGCTGTCCCG
*ureB*	GGAATGGTTTGAAACGAGGA	CCGCTTCCACTAACCCCACTA
*ureE*	TCTTGGCTTGGATGTGAATG	GGAATGGTTTGAAACGAGGA
*ureF*	GGGGCTTGTGGATAGCATAA	CGCATTCTTTTGGGCTAGAA
*ureH*	AGATCCCAAAACCACCGACTT	ACGGCTCCATCCACTCCTT
*nixA*	CATATGATCATAGAGCGTTTAATG	CTCGAGTTTCATGACCACTTTAAA

#### 2.5.6 Urease activity assay

The method established by Debowski et al. (2017) was used to evaluate the effect of drugs on *H. pylori* urease activity. Mature *H. pylori* cultures were adjusted to a turbidity of 1.0 McFarland. Bacterial suspension and drug solutions (final concentrations of 2 × MIC, 1 × MIC, and 0.5 × MIC) were mixed at a 1:1 ratio in six-well plates. A growth control group and a positive control group (acetohydroxamic acid, 40 μg/mL) were also established. Cultures were incubated under microaerophilic conditions at 37 °C with shaking for 24 h. The sediments were collected by centrifugation at 4 °C and washed three times with cold PBS. To ensure consistent bacterial counts, all groups were adjusted to OD₆₀₀ = 0.2, then diluted with PBS containing 0.2% Tween-20 at a 1:1 volume ratio. An appropriate volume of the diluted bacterial suspension was mixed with phenol red solution (pH 6.8, 25 mM phosphate buffer, 250 μM phenol red), and incubated at a constant temperature for 5 min. Finally, a fixed volume of 0.5 M urea solution was added, and the absorbance at 560 nm was recorded using a microplate reader every 72 s for a total of 30 cycles. Urease activity was quantified by the rate of change in absorbance at 560 nm (ΔA/min) and expressed as a percentage relative to the *H. pylori* growth control group. Each experiment was repeated three times.

#### 2.5.7 Scanning electron microscope

A Sigma 500 scanning electron microscope (ZEISS, Germany) was used to observe morphological and ultrastructural changes in *H. pylori* after drug treatment (Shen et al., 2021). Growth control and drug-treated groups were prepared as described in section 2.5.5. Sediments were collected by centrifugation at 4 °C and washed three times with PBS, then fixed overnight at low temperature in 2.5% glutaraldehyde. Samples underwent gradient dehydration with ethanol and tert-butanol substitution, followed by drying at 60 °C. After metal coating, bacterial morphology was observed under appropriate magnification.

#### 2.5.8 Effect on CagA protein expression

Western blotting was used to assess the effect of drug treatment on the expression of *H. pylori* virulence protein CagA. Mature SS1 strains were adjusted to 1.0 McFarland and mixed in equal volumes with BHI medium containing 20% FBS and drug solutions (final concentrations of MIC and 0.5 × MIC) in six-well plates to establish control and treatment groups. Cultures were incubated under microaerophilic conditions at 37 °C with shaking for 24 h. Bacterial pellets were collected by centrifugation at 4 °C and washed three times. Each sample was lysed with 80 μL of RIPA buffer (containing 0.1% protease inhibitors and 1% phosphatase inhibitors) on ice for 20 min. Protein concentrations were measured using a BCA assay kit and normalized. The diluted protein samples were separated by SDS-PAGE, transferred to PVDF membranes, and blocked with 5% skim milk at room temperature for 2 h. Membranes were incubated overnight at 4 °C with a primary anti-*H. pylori* CagA antibody. After washing, membranes were incubated with a mouse-derived secondary antibody (1:2500 dilution) for 1 h at room temperature. Protein bands were visualized using the BeyoECL Plus Kit and imaged with the ChemiScope 6,200 system (Bio-Rad, Hercules, CA, United States). Grayscale intensities of protein bands were semi-quantified using ImageJ software.

### 2.6 Metabolomics analysis

An untargeted metabolomics approach based on high-performance liquid chromatography–mass spectrometry (HPLC-MS) was used to assess changes in *H. pylori* metabolism before and after drug treatment (Yap et al., 2017). Mature SS1 strains were adjusted to 1.0 McFarland and cultured under microaerophilic conditions at 37 °C with shaking for 24 h. After treatment with or without drug, incubation continued for another 12 h. Bacteria were collected by centrifugation at 4 °C, washed three times, and quenched with pre-chilled methanol. After centrifugation at 4 °C, the supernatant was discarded and the pellet was treated with pre-chilled methanol–water solution (1:1, v/v). Samples were ultrasonicated at 35% amplitude for 10 min and centrifuged again at 4 °C. The resulting supernatant was immediately stored at −80 °C for subsequent HPLC-MS analysis.

The liquid chromatography (LC) analysis was conducted using a Hypersil GOLD column (100 mm × 2.1 mm, 1.9 μm) maintained at 40 °C with a flow rate of 0.2 mL/min. For positive ion mode, the mobile phase consisted of 0.1% formic acid in water (A) and methanol (B), while negative ion mode utilized 5 mM ammonium acetate (pH 9.0, A) and methanol (B). Gradient elution was applied under both ionization modes. Mass spectrometry (MS) conditions included a mass scan range of m/z 100–1,500, an electrospray ionization (ESI) source operated at 3.5 kV, and a dual polarity mode (positive/negative). Gas parameters were set to 35 L/min sheath gas, 10 L/min auxiliary gas, and an auxiliary gas heater temperature of 350 °C. The ion transfer tube and ion funnel were maintained at 320 °C and RF level 60 V, respectively. Data-dependent MS/MS acquisition was employed to automatically trigger fragmentation of precursor ions based on real-time spectral intensity. All parameters were optimized to ensure high-resolution detection and accurate identification of analytes. Deconvoluted chromatographic data were integrated and metabolites were identified using the Fiehn and NIST2017 databases (Probability ≥ 50 or match value ≥ 750) (Huang et al., 2016). Principal Component Analysis (PCA) and Partial Least Squares Discriminant Analysis (PLS-DA) were performed using R-4.4.3. Differential metabolites were screened with thresholds of *p* < 0.05 and |log₂FC| > 1, and visualized with heatmaps and volcano plots. The KEGG database was used for metabolite and pathway annotation, and MetaboAnalyst 6.0^1^ was used for pathway enrichment analysis.

### 2.7 Network pharmacology and molecular docking

#### 2.7.1 Prediction of potential targets of NBP

The SMILES structure of NBP was retrieved from the PubChem database^2^ and imported into the SwissTargetPrediction platform.^3^ The species was set to “*Homo sapiens*” and targets were selected with Probability > 0.05 (Zhang J. et al., 2022).

#### 2.7.2 Screening of *Helicobacter pylori*-related targets

*Helicobacter pylori* infection-related targets were obtained by querying “*H. pylori* infection” in OMIM,^4^ DisGeNET,^5^ and GeneCards.^6^ Filtering criteria were set as follows: “relevance score” ≥ median (GeneCards) and “score gda” > 0.1 (DisGeNET) (Li et al., 2023).

#### 2.7.3 Identification of common targets between NBP and *Helicobacter pylori*

Common targets between NBP and *H. pylori* infection were identified using the Venny 2.1.0 tool^7^ and visualized with a Venn diagram.

#### 2.7.4 PPI network construction and core target screening

The intersecting targets were imported into the STRING database,^8^ with the “multiple proteins” mode selected and species set to *Homo sapiens*. PPI data were downloaded and visualized using Cytoscape 3.9.1 software. The CentiScaPe 2.2 plugin was used to calculate topological parameters such as degree centrality, closeness centrality, and betweenness centrality, for core target identification and PPI network construction.

#### 2.7.5 GO and KEGG enrichment analysis

The common targets of NBP and *H. pylori* were imported into the Metascape database,^9^ with species limited to *Homo sapiens*. GO and KEGG pathway enrichment analyses were performed, and the results were visualized using the online tool Bioinformatics.^10^

#### 2.7.6 Molecular docking

The 3D structures of core target proteins were downloaded from the PDB database^11^ and imported into AutoDock 1.5.7 for molecular docking. The optimal docking results were visualized using Python and PyMOL.

### 2.8 Data statistics and analysis

All data were statistically analyzed using GraphPad Prism 8.0.2. Experimental results are presented as mean ± standard deviation (X ± SD). Differences between groups were assessed using one-way ANOVA, with significance tested using *post hoc* multiple comparison methods. *p < 0.05 was considered statistically significant.

## 3 Results

### 3.1 Preparation and characterization of NBP-NPs

**Figure 1:**
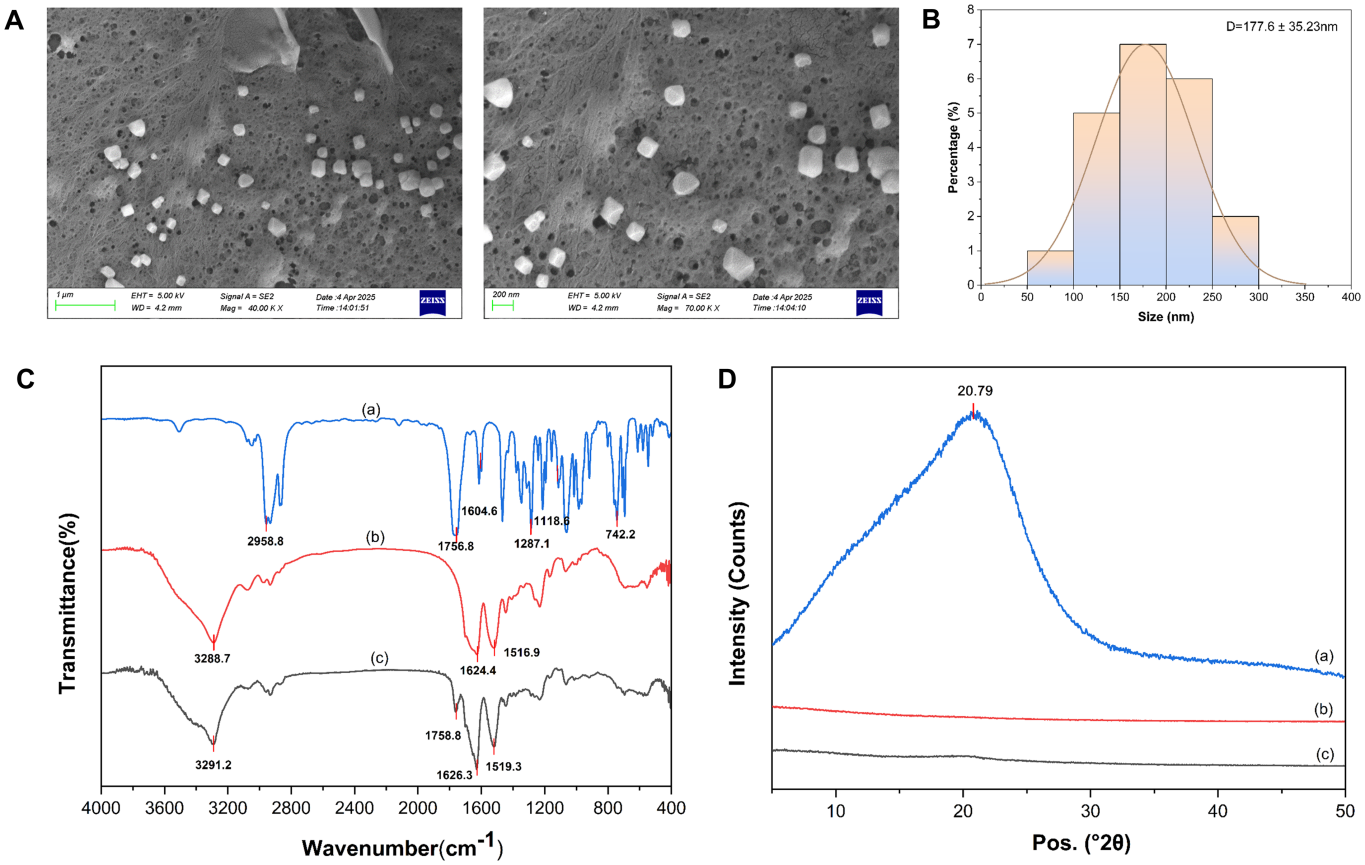
Characterization of NBP-NPs. **(A)** Representative SEM images of NBP-NPs at magnifications of 40.0 kX and 70.0 kX. **(B)** Corresponding particle size distribution histogram. Data are presented as mean ± standard deviation. **(C)** FT-IR spectra of the samples in the range of 4,000–400 cm^−1^. **(D)** XRD patterns of the samples in the 2θ range of 5°–50°. Curves **(A)**, **(B)**, and **(C)** represent NBP, SF-NPs, and NBP-NPs, respectively.

RSF solution is a kinetically stable yet thermodynamically unstable system. When subjected to external stimuli such as pH changes, elevated temperature, metal ions, organic solvents, or mechanical stress, the fibroin molecular chains can be induced to aggregate in an ordered fashion. This leads to a transformation of its secondary structure into *β*-sheet conformations, ultimately forming dense and rigid microcrystalline structures (Hu et al., 2011; Terada et al., 2016). Notably, these ordered structures can undergo reversible transitions under specific environmental conditions (e.g., catalysis, acidity, stress, or other factors), reverting to a flexible random coil conformation (Chen R. et al., 2019; Gou et al., 2019; Seib et al., 2013). In this study, nanoparticles were prepared via a desolvation method. Under the induction of acetone, an organic solvent, silk fibroin rapidly dispersed, and hydrophobic interactions between the hydrophobic domains triggered molecular aggregation, promoting colloid formation and further self-assembly into spherical nanoparticles. The particle size and zeta potential of nanoparticles were determined by dynamic light scattering (DLS), with results summarized in Table 2. SF-NPs exhibited an average hydrodynamic diameter of 78.6 ± 0.6 nm (PDI = 0.166), while NBP-NPs showed a diameter of 227.6 ± 1.3 nm (PDI = 0.215). The corresponding zeta potentials were −24.3 ± 0.7 mV and −25.2 ± 1.6 mV, indicating favorable kinetic stability of the system. SEM images and corresponding particle size distribution histograms of NBP-NPs are presented in Figures 1A,B. The fabricated nanoparticles exhibited a near-spherical morphology, with a mean particle size of 177.6 ± 35.23 nm as determined by SEM analysis. This value is larger than the hydrodynamic diameter measured by DLS, consistent with the dehydration-induced shrinkage during sample preparation.

**Table 2:** Particle size, PDI, and zeta potential of NBP-NPs.

Nanoparticles	Size (nm)	PDI	Zeta Potential(mV)
SF-NPs	78.6 ± 0.6	0.166	−24.3 ± 0.7
NBP-NPs	227.6 ± 1.3	0.215	−25.2 ± 1.6

During the desolvation process, silk fibroin molecular chains undergo stretching and alignment-driven rearrangement, with a secondary structure transition from random coils to *β*-sheets. This results in the formation of stable microcrystalline domains within the nanoparticles. Such structural evolution facilitates efficient encapsulation of active drug molecules into the hydrophobic core of the nanoparticles, yielding an encapsulation efficiency of 45.80 ± 1.5% and drug loading content of 15.27 ± 0.8% for NBP.

Fourier Transform Infrared Spectroscopy (FT-IR) spectroscopy, known for its high sensitivity and molecular specificity, is a key analytical tool to characterize polymer–drug interactions (Chen Y. et al., 2019). Figure 1C displays the FT-IR spectra of pristine NBP, SF-NPs, and NBP-NPs. NBP exhibited the following characteristic absorption peaks: a prominent ester carbonyl (-COO-) stretching vibration at 1750 cm^−1^, a typical out-of-plane bending vibration of monosubstituted benzene rings at 745 cm^−1^, and stretching vibrations of aliphatic C-H bonds (-CH₂, -CH₃) in the 2,850–3,000 cm^−1^ range. Additionally, a peak at 1600 cm^−1^ corresponds to the benzene ring skeletal (C=C) vibration, while peaks at 1280 cm^−1^ and 1,120 cm^−1^ correspond to asymmetric and symmetric C–O–C ester bond stretching modes, respectively. Compared to pristine NBP, both SF-NPs and NBP-NPs showed significantly different spectral features. Both nanoparticle systems exhibited a distinct amide I band absorption peak at 1620 cm^−1^ (attributed to C=O stretching) and an amide II band at 1520 cm^−1^ (primarily from N–H bending vibrations). Importantly, a weak absorption peak near 1750 cm^−1^ was detected in the NBP-NPs, matching the ester carbonyl peak of pristine NBP, suggesting that trace amounts of pristine NBP may be adsorbed on the nanoparticle surface, implying weak interactions between NBP and the nanoparticles.

X-ray Diffraction (XRD) spectra of pristine NBP, SF-NPs, and drug-loaded nanoparticles are shown in Figure 1D. NBP displayed typical semi-crystalline characteristics, with a broad peak near 2θ = 20°. In contrast, both SF-NPs and NBP-NPs exhibited amorphous features. Notably, the broad peak nearly disappeared upon NBP loading into the silk fibroin (SF) matrix, indicating strong molecular interactions during the encapsulation process. Combined with FT-IR analysis, the interaction between NBP and SF is hypothesized to primarily occur through two distinct mechanisms. First, hydrogen bonding forms between the ester carbonyl group of NBP and the amide functional groups within the SF structure, as evidenced by characteristic vibrational band shifts in FT-IR spectra (Wu et al., 2024). Second, hydrophobic interactions arise from the intercalation of NBP’s aromatic ring system into the *β*-sheet domains of SF, driven by complementary molecular geometry and nonpolar affinity.

### 3.2 *In vitro* antibacterial and bactericidal activity of NBP and its nanoparticles

#### 3.2.1 MIC and MBC

**Figure 2:**
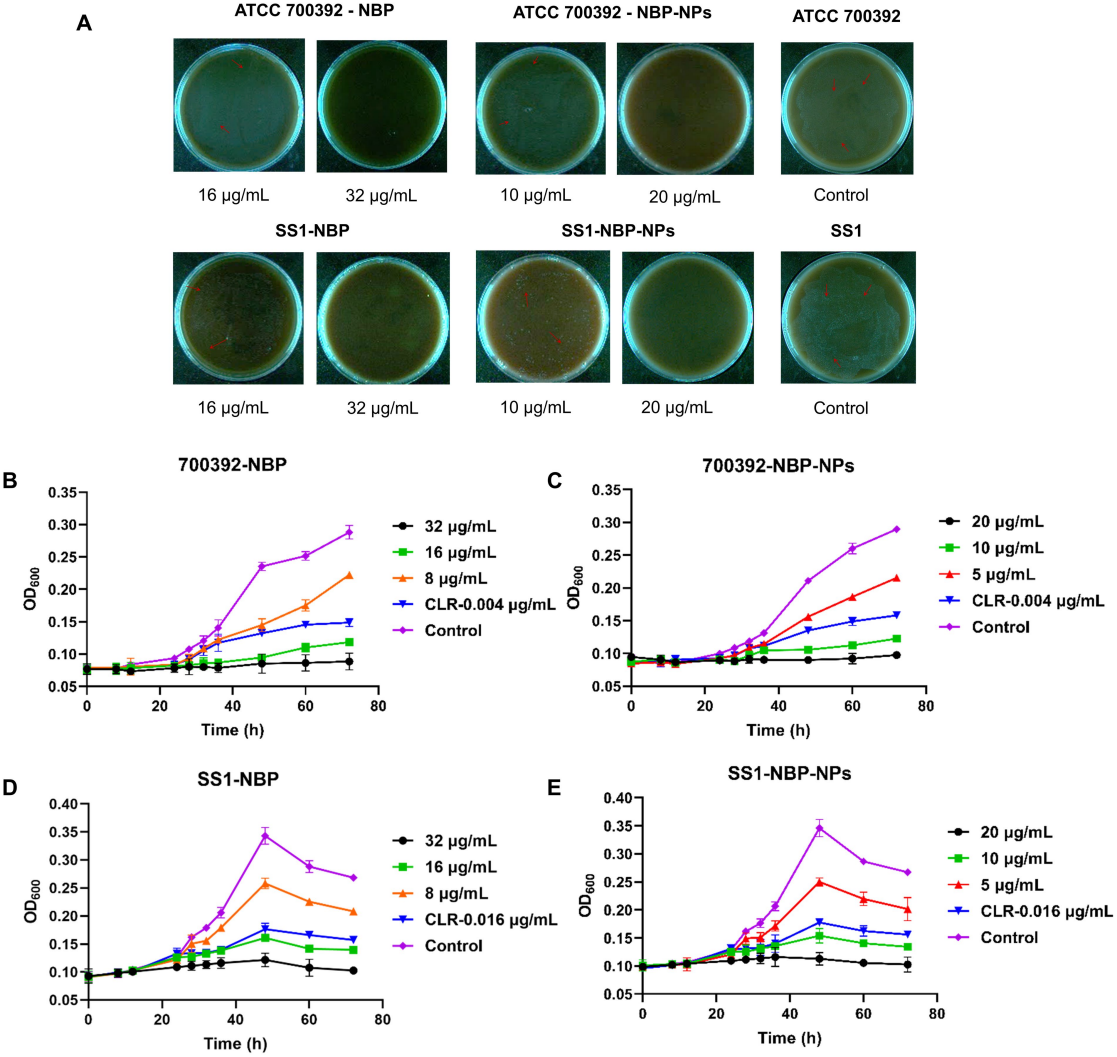
Inhibitory and bactericidal effects of NBP and NBP-NPs. **(A)** The colony morphology of *H. pylori* strains after treatment with various drug concentrations. **(B)** Bacterial growth inhibition kinetics of strain ATCC 700392 following treatment with NBP. **(C)** Bacterial growth inhibition kinetics of strain ATCC 700392 following treatment with NBP-NPs. **(D)** Bacterial growth inhibition kinetics of strain SS1 following treatment with NBP. **(E)** Bacterial growth inhibition kinetics of strain SS1 following treatment with NBP-NPs.

This study determined the MICs and MBCs of NBP and NBP-NPs against different *H. pylori* strains (Table 3), and the colony morphology of *H. pylori* strains ATCC 700392 and SS1 after treatment with various drug concentrations was shown in Figure 2A. The results showed that the MICs of NBP against standard strains ranged from 8 to 16 μg/mL, and MBCs ranged from 16 to 32 μg/mL. For clinical strains, MICs ranged from 4 to 16 μg/mL and MBCs from 8 to 32 μg/mL. NBP exhibited both inhibitory and bactericidal effects against all tested strains, with the strongest activity against clinical isolates QYZ001, QYZ003, and QYZ004. All tested strains had an MBC/MIC ratio of 2.0, indicating a strong bactericidal effect. Notably, NBP-NPs demonstrated enhanced anti-*H. pylori* activity compared to NBP, as evidenced by a reduced MIC. For example, the MIC for the standard strain ATCC 700392 was 10 μg/mL for the nanoparticles, which is a 37.5% reduction compared to the MIC of NBP. Furthermore, the blank nanoparticle carrier at a concentration of 1 mg/mL showed no inhibitory effect, confirming the advantage of the nanoparticles in enhancing antibacterial activity.

**Table 3:** The MIC and MBC results of NBP and NBP-NPs on different *H. pylori* strains.

Sample	*H. pylori* strains	Drug sensitivity	MIC (μg/ml)	MBC (μg/ml)
NBP	ATCC 700392	S	16	32
	ATCC 43504	R (MTZ)	8	16
	SS1	S	16	32
	CS01	R (CLR)	16	32
	QYZ-001	R (MTZ)	4	8
	QYZ-003	R (CLR, MTZ, LEF)	4	8
	QYZ-004	R (CLR, MTZ, LEF, AMO)	4	8
NBP-NPs	ATCC 700392	S	10	20
	ATCC 43504	R (MTZ)	5	10
	SS1	S	10	20
	CS01	R (CLR)	10	20
	QYZ-001	R (MTZ)	2.5	5
	QYZ-003	R (CLR, MTZ, LEF)	2.5	5
	QYZ-004	R (CLR, MTZ, LEF, AMO)	2.5	5

The susceptibility of the *H. pylori* strains (reference strains ATCC 700392 and ATCC 43504; clinical strains SS1, CS01, QYZ-001, QYZ-003, and QYZ-004) to clarithromycin (CLR), metronidazole (MTZ), levofloxacin (LEF), and amoxicillin (AMO) was assessed against EUCAST (2019) breakpoints.

#### 3.2.2 Inhibiting kinetics assay

As shown in Figures 2B–E, both NBP and NBP-NPs exhibited clear inhibitory effects on the standard strain ATCC 700392 and clinical strain SS1. ATCC 700392 showed rapid growth between 24 and 48 h, whereas treatment with MIC concentrations of NBP or NBP-NPs kept colony counts stable within 48 h, indicating a strong inhibitory effect superior to the positive control. At 2×MIC, bacterial growth was nearly completely inhibited, while the 0.5×MIC concentration only delayed growth without significantly reducing colony counts. Similar trends were observed in the SS1 strain. Overall, NBP exhibited potent and stable inhibitory potential at MIC or higher concentrations, and NBP-NPs demonstrated enhanced activity.

#### 3.2.3 No antagonism in combination with antibiotics

Drug synergy tests showed additive or irrelevant effects (FICI = 0.625–1.25) when NBP was combined with four clinically used antibiotics, without any antagonistic interactions (Table 4). Further analysis revealed significant strain-specific differences: NBP combined with metronidazole (MTZ) showed the best inhibition against ATCC 700392, while the combination with levofloxacin (LEF) was most effective against the SS1 clinical isolate. Notably, some FICI values approached the threshold for synergy (FICI ≤ 0.5), suggesting potential synergistic antibacterial effects.

**Table 4:** FICI index analysis of NBP and NBP-NPs combined with clinical antibiotics.

*H. pylori* strains	Combination		MIC (μg/ml)			FICI	Interaction
	Drug	Antibiotics	Drug	Antibiotics	Combination		
ATCC 700392	NBP	CLR	16	0.004	16+0.001	1.25	Irrelevant
		MTZ	16	1.6	8+0.2	0.625	Additive
		LEF	16	0.8	8+0.2	0.75	Additive
		AMO	16	0.1	8+0.025	0.75	Additive
	NBP-NPs	CLR	10	0.004	10+0.001	1.25	Irrelevant
		MTZ	10	1.6	5+0.2	0.625	Additive
		LEF	10	0.8	5+0.2	0.75	Additive
		AMO	10	0.1	5+0.025	0.75	Additive
SS1	NBP	CLR	16	0.016	8+0.004	0.75	Additive
		MTZ	16	1.6	8+0.8	1	Additive
		LEF	16	1.6	8+0.2	0.625	Additive
		AMO	16	0.4	16+0.1	1.25	Irrelevant
	NBP-NPs	CLR	10	0.016	5+0.004	0.75	Additive
		MTZ	10	1.6	5+0.8	1	Additive
		LEF	10	1.6	5+0.2	0.625	Additive
		AMO	10	0.4	10+0.1	1.25	Irrelevant

#### 3.2.4 RT-qPCR for detection of virulence gene expression

**Figure 3:**
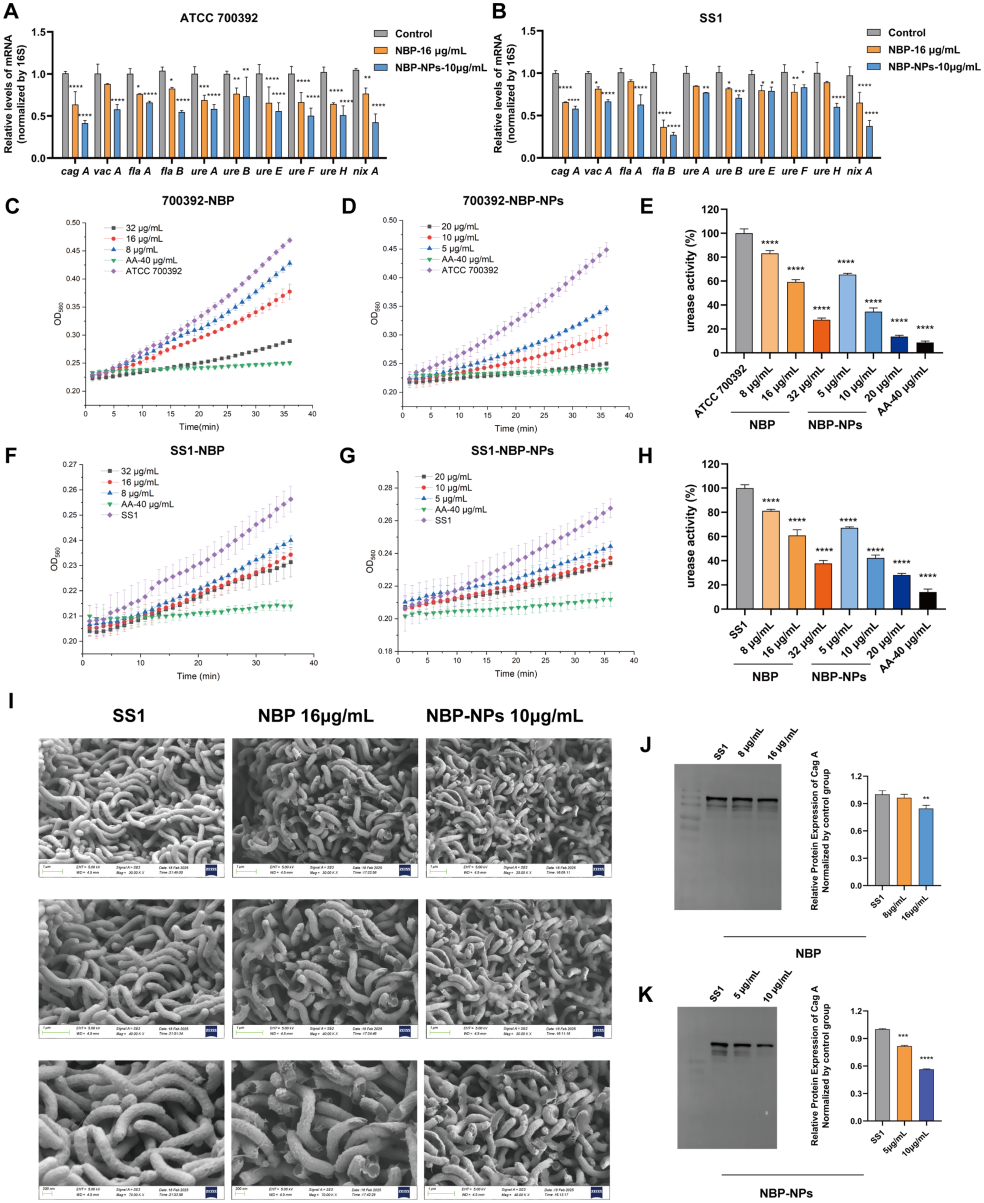
Molecular mechanisms of drug action against *H. pylori*. **(A)** Impact of NBP and NBP-NPs treatment on virulence gene expression levels in ATCC 700392. **(B)** Impact of NBP and NBP-NPs treatment on virulence gene expression levels in SS1. **(C–E)** Effect of drug treatment on urease activity in ATCC 700392. (F–H) Effect of drug treatment on urease activity in SS1 (*n* = 3, *p < 0.05, **p < 0.01, ***p < 0.001, ****p < 0.0001 vs. control group). **(I)** Drug-induced ultrastructural alterations in *H. pylori* SS1 observed by SEM (30.0 kx, 40.0 kx, 70.0 kx). CagA protein expression in *H. pylori* SS1 treated with **(J)** NBP and **(K)** NBP-NPs (*n*=3, *p < 0.05, **p < 0.01, ***p < 0.001, ****p < 0.0001 vs. con trol group).

RT-qPCR results (Figures 3A,B) showed that both NBP and NBP-NPs downregulated the expression of key *H. pylori* virulence genes, with varying effectiveness across strains and genes. NBP more strongly suppressed *cagA* and urease genes (*ureE–H*) in the ATCC 700392 strain (inhibition rate: 33.59–36.49%), while it more effectively inhibited the flagellar gene *flaB* in SS1 (63.51 ± 8.2%). Notably, the nanoparticles exhibited a stronger gene-silencing effect. For example, suppression of the *nixA* gene was improved by 27.46–34.03% compared to NBP.

#### 3.2.5 Urease activity assay

The inhibitory effects of different concentrations of NBP and NBP-NPs on *H. pylori* urease activity are shown in Figures 3C–H. A known urease inhibitor (AA, 40 μg/mL) was used as a positive control. Both NBP and NBP-NPs exhibited dose-dependent urease inhibition, with greater efficacy against the standard strain ATCC 700392. These phenotypic findings align with molecular results from RT-qPCR, where NBP and NBP-NPs downregulated the transcription of urease-related genes (*ureA–B*, *ureE–H*). Again, the nanoparticles showed stronger inhibitory effects, likely due to enhanced drug delivery efficiency.

#### 3.2.6 Morphological alterations in *Helicobacter pylori* revealed by SEM

SEM was used to observe morphological changes in *H. pylori* treated with MIC concentrations of NBP and NBP-NPs (Figure 3I). In the control group, bacteria maintained a characteristic helical shape with smooth surfaces and intact membranes. In contrast, treated groups showed evident morphological damage, including ruptured membranes, cellular deformation, and collapse at polar ends with cytoplasmic leakage. These results suggest that the antibacterial activity may be associated with disruption of bacterial membrane integrity.

#### 3.2.7 Western blot analysis of CagA protein expression

Western blot results (Figures 3J,K) showed that both NBP and its nanoparticles significantly downregulated the expression of the *H. pylori* SS1 virulence protein CagA. Quantitative analysis indicated that NBP inhibited CagA expression by 84.52 ± 2.8% at MIC concentration, while NBP-NPs achieved even greater suppression at 56.50 ± 1.3%.

### 3.3 Metabolomics study

**Figure 4:**
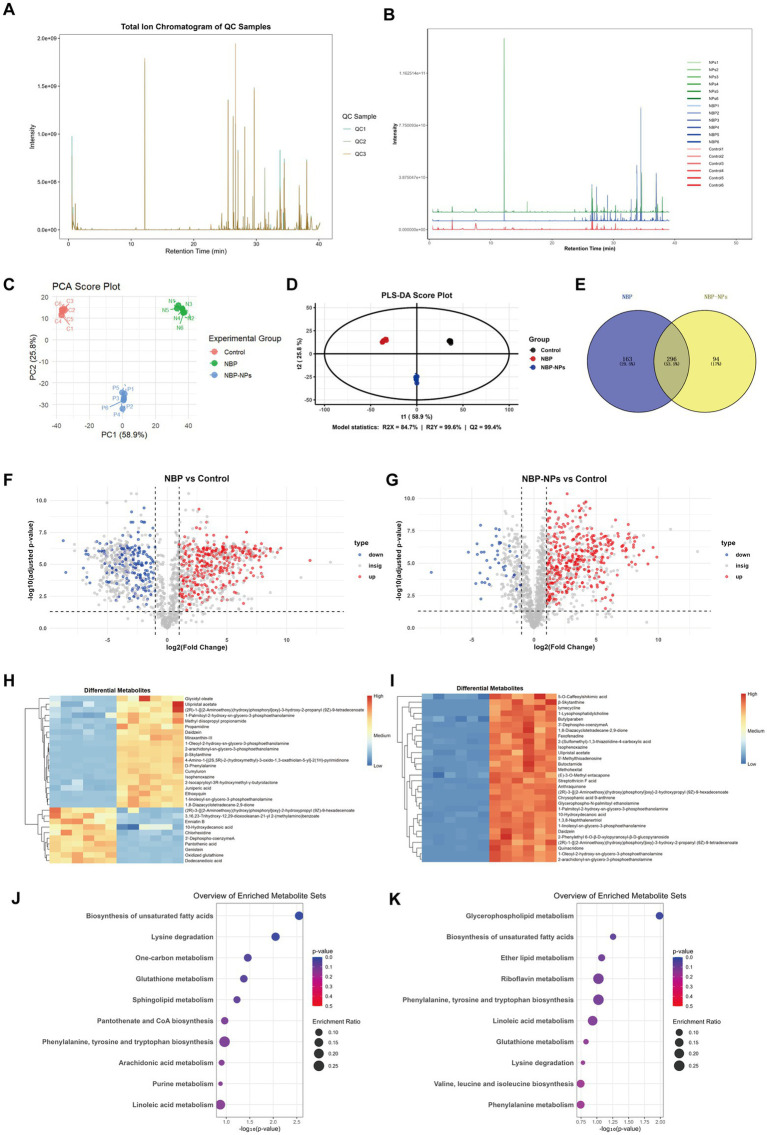
Metabolite analysis of *H. pylori* SS1 treated *in vitro* with NBP and its nanoparticles. **(A)** TIC of QC sample chromatogram. **(B)** TIC of bacterial metabolites. The vertical axis represents the normalized intensity offset index for each group. The three distinct colors correspond to the three different experimental groups. **(C)** PCA of bacterial metabolites. **(D)** PLS-DA of bacterial metabolites. **(E)** Venn diagram analysis of differential metabolites between the NBP group and the NBP-NPs group. **(F)** Volcano plot depicting differential metabolites in the NBP group versus the control. **(G)** Volcano plot depicting differential metabolites in the NBP-NPs group versus the control. Metabolites marked in red, green, and gray represent those that are significantly upregulated, downregulated, or show no significant change, respectively. **(H)** Clustered heatmap showing the metabolite profiles of the NBP group compared to the control. **(I)** Clustered heatmap showing the metabolite profiles of the NBP-NPs group compared to the control. The color bar represents the relative abundance level of metabolites, with gradients from blue (low) to red (high). **(J)** KEGG pathway enrichment analysis of metabolites from *H. pylori* SS1 after treatment with NBP. **(K)** KEGG pathway enrichment analysis of metabolites from *H. pylori* SS1 after treatment with NBP-NPs. The size of each circle corresponds to the number of metabolites mapped to the pathway, and the color depth reflects the significance of the enrichment.

Studies have shown that the impact of drugs on bacterial metabolism and physiological functions is a key factor in bacterial death (Zheng et al., 2023). In this study, non-targeted metabolomics based on HPLC-MS technology was used to investigate the effects of drug treatment on the metabolites of *H. pylori*. A total of 1,549 metabolites were identified through databases including mzCloud, mzVault, and Masslist. These metabolites included classes such as glycerophospholipids, fatty acids, carbohydrates, amino acids, and nucleotides. Overall, the 1,549 metabolites were diverse and evenly distributed. The QC sample chromatogram and total ion chromatogram (TIC) are shown in Figures 4A,B, where the metabolite baseline was stable, separation was efficient, and most adjacent peaks had a resolution greater than 1.5. No significant co-elution was observed. The retention times were highly reproducible and covered a broad range of metabolite classes, from polar small molecules to nonpolar lipids. PCA and PLS-DA analyses were performed on the metabolites of *H. pylori* SS1 treated with NBP, the nanoparticles, and the control group. The PCA revealed that the cumulative variance explained by the first two principal components was 84.7% (PC1 = 58.9%, PC2 = 25.8%), indicating a clear separation among the three groups (Figure 4C). A classification model was constructed using PLS-DA combined with permutation testing (Yu et al., 2023). The data were preprocessed by Z-score normalization, and model reliability was confirmed (*R*^2^X = 84.7%, *R*^2^Y = 99.6%, *Q*^2^ = 99.4%). These results were consistent with PCA, both successfully distinguishing the metabolic profiles of *H. pylori* post-treatment (Figure 4D).

Volcano plots were constructed based on the fold change (FC) threshold (|log₂FC| > 1) and significance threshold (*p* < 0.05). As shown in Figures 4F,G, red and blue dots represent upregulated and downregulated metabolites, respectively. In the NBP group, 459 differential metabolites were identified compared to the control (297 upregulated, 162 downregulated), while 390 differential metabolites were found in the nanoparticles group (350 upregulated, 40 downregulated). A Venn diagram analysis of these differential metabolites (Figure 4E) revealed 296 overlapping metabolites, indicating that nearly half of the differential metabolites were shared between the NBP and NBP-NPs groups.

Further screening was conducted to identify significant differential metabolites with Variable Importance in Projection (VIP > 1) and adjusted (*p* < 0.05). The top 30 key metabolites were extracted in descending order (Table 5). It was evident that metabolites such as anthraquinone, chrysophanic acid 9-anthrone, cyclo (L-leucyl-L-prolyl), and 5′-methylthioadenosine showed no significant changes in the NBP group compared to the control but were markedly upregulated in the nanoparticles group. Furthermore, although phospholipid compounds such as 1-oleoyl-2-hydroxy-sn-glycero-3-PE were upregulated in both treatment groups, the fluctuations in the nanoparticles group were much greater than in the NBP group.

**Table 5:** List of the top 30 differential metabolites.

No.	Metabolites	VIP
1	Anthraquinone	1.39360135
2	Glycerophospho-N-palmitoyl ethanolamine	1.39299311
3	Chrysophanic acid 9-anthrone	1.39264498
4	Silodosin	1.39174063
5	1-Palmitoyl-2-hydroxy-sn-glycero-3-PE	1.39144125
6	Cyclo(L-leucyl-L-prolyl)	1.39037763
7	5′-Methylthioadenosine	1.38927238
8	lysophosphatidylcholine	1.38888993
9	1-Oleoyl-2-hydroxy-sn-glycero-3-PE	1.38862763
10	2-arachidonyl-sn-glycero-3-phosphoethanolamine	1.38850657
11	Streptothricin F acid	1.38834391
12	3’-Dephospho-coenzymeA	1.38800416
13	10-Hydroxydecanoic acid	1.38773156
14	Bevenopran	1.38565903
15	butoctamide	1.38556587
16	Quinacridone	1.38538539
17	1,3,8-Naphthalenertriol	1.38535963
18	Lymecycline	1.38530737
19	1-linoleoyl-sn-glycero-3-phosphoethanolamine	1.38522154
20	Methohexital	1.38484436
21	Daidzein	1.38443672
22	2-Phenylethyl 6-O-β-D-xylopyranosyl-β-D-glucopyranoside	1.38425429
23	Diftalone	1.38279634
24	Isophenoxazine	1.38233428
25	(E)-3-O-Methyl entacapone	1.38149112
26	5-Methoxyindole acetate	1.37654674
27	5-O-Caffeoylshikimic acid	1.37426017
28	Ulipristal acetate	1.37348486
29	β-Skytanthine	1.37281952
30	1,8-Diazacyclotetradecane-2,9-dione	1.3714671

Cluster heatmap analysis was performed for the top 30 up- and downregulated metabolites in both the NBP and nanoparticles groups (Zheng et al., 2023; Figures 4H,I). The direction of metabolic reprogramming was highly similar between the two groups, but the nanoparticles group exhibited more pronounced fluctuations in metabolite levels.

KEGG and MetaboAnalyst pathway enrichment analysis explored the biological significance of these metabolites for *H. pylori*. As shown in Figures 4J,K, five significantly enriched pathways (*p* < 0.05) were identified, including Biosynthesis of unsaturated fatty acids, Lysine degradation, One-carbon metabolism, Glutathione metabolism and Glycerophospholipid metabolism. These findings suggest that NBP may exert its antibacterial effects *in vitro* by disrupting bacterial membrane integrity, blocking energy supply, and interfering with signal transduction.

### 3.4 Network pharmacology and molecular docking

**Figure 5:**
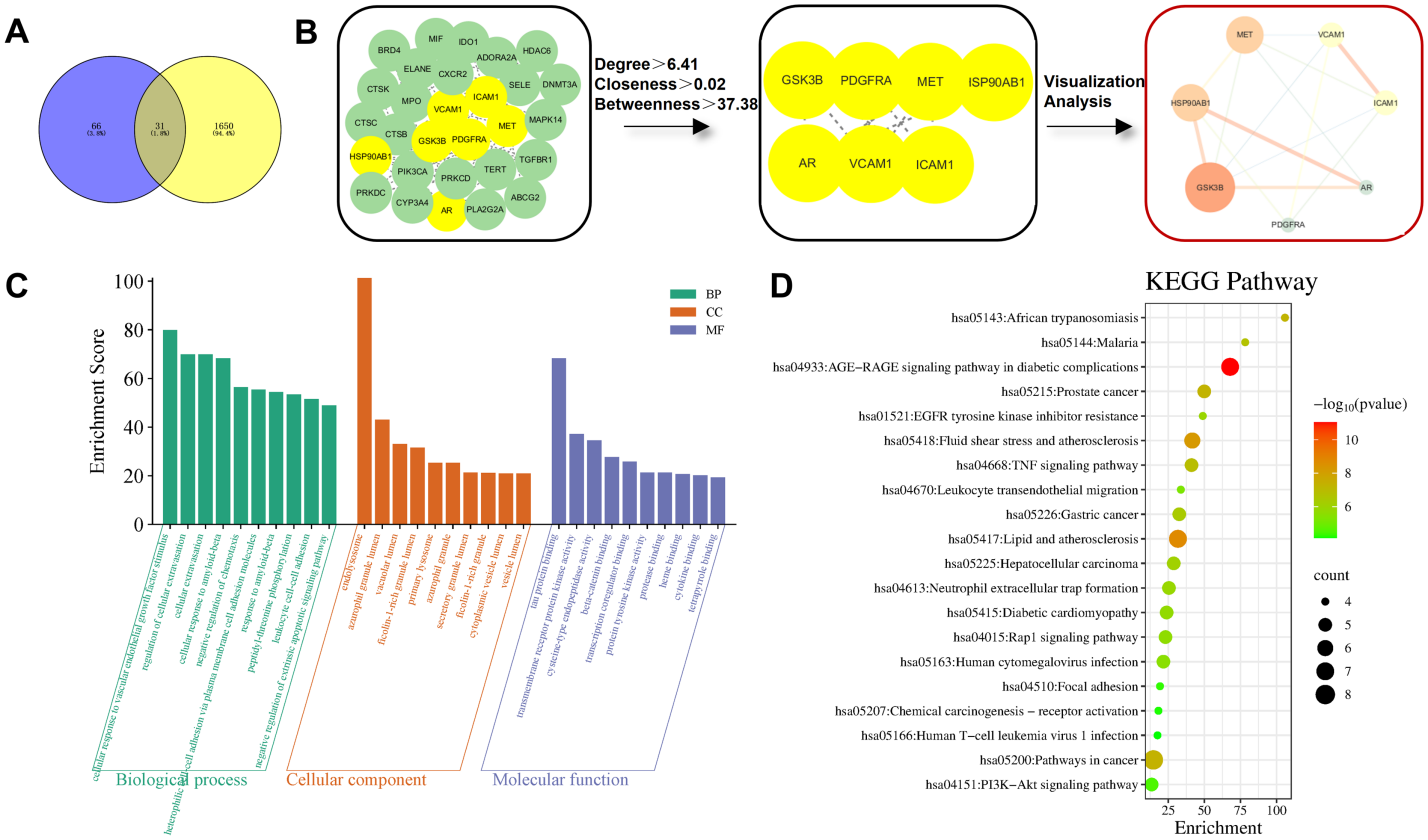
Network pharmacology analysis of NBP against *H. pylori* infection. **(A)** Venn diagram of drug targets and *H. pylori* infection-related targets. Blue indicates drug targets; yellow indicates disease targets. **(B)** The protein–protein interaction (PPI) network visualizes core targets, where the size of each node (circle) is proportional to its connectivity degree, highlighting topologically important hubs. **(C)** GO enrichment analysis of common targets; **(D)** KEGG pathway enrichment analysis of common targets. The size of a bubble represents the number of enriched targets, and the color depth reflects the significance of the enrichment.

Database screening yielded 97 drug targets and 1,681 *H. pylori* infection-related targets. A Venn diagram analysis showed 31 common targets (Figure 5A). These intersection targets were subjected to topological analysis and used to construct a PPI network (Figure 5B). Nodes with higher degree values in the network suggest more significant regulatory roles in protein–protein interactions.

Seven core targets were identified: Glycogen synthase kinase 3 beta (*GSK3B*), Heat shock protein 90 alpha family class B1 (*HSP90AB1*), Mesenchymal-Epithelial Transition factor (*MET*), Vascular cell adhesion molecule 1 (*VCAM1*), Intercellular adhesion molecule 1 (*ICAM1*), Androgen receptor (*AR*), and Platelet-derived growth factor receptor alpha (*PDGFRA*). Among these, *GSK3B* and *HSP90AB1* acted as central hub nodes and may play key regulatory roles in *H. pylori* infection.

GO enrichment analysis of the 31 common targets yielded 321 entries: 261 for biological processes (BP), 33 for cellular components (CC), and 27 for molecular functions (MF). The top 10 entries in each category were visualized (Figure 5C). BP terms primarily included regulation of inflammation, angiogenesis, stress response, cell migration, and signal transduction. CC terms involved lysosomal structures, secretory granule lumen, and vesicle transport systems. MF terms included kinase activity regulation and protein binding. Further KEGG enrichment analysis identified 79 pathways. The top 20 were visualized (Figure 5D). These targets were significantly enriched in core pathways such as inflammation and immune regulation, cancer progression, metabolic disorders, and signal transduction networks. This suggests a possible mechanism where NBP exerts its therapeutic effects through multi-target synergy, inhibiting *H. pylori*-mediated inflammatory cascades, oncogenic signaling, and metabolic disruption, thereby intervening in infectious gastritis, precancerous lesions, and systemic pathological processes.

**Figure 6:**
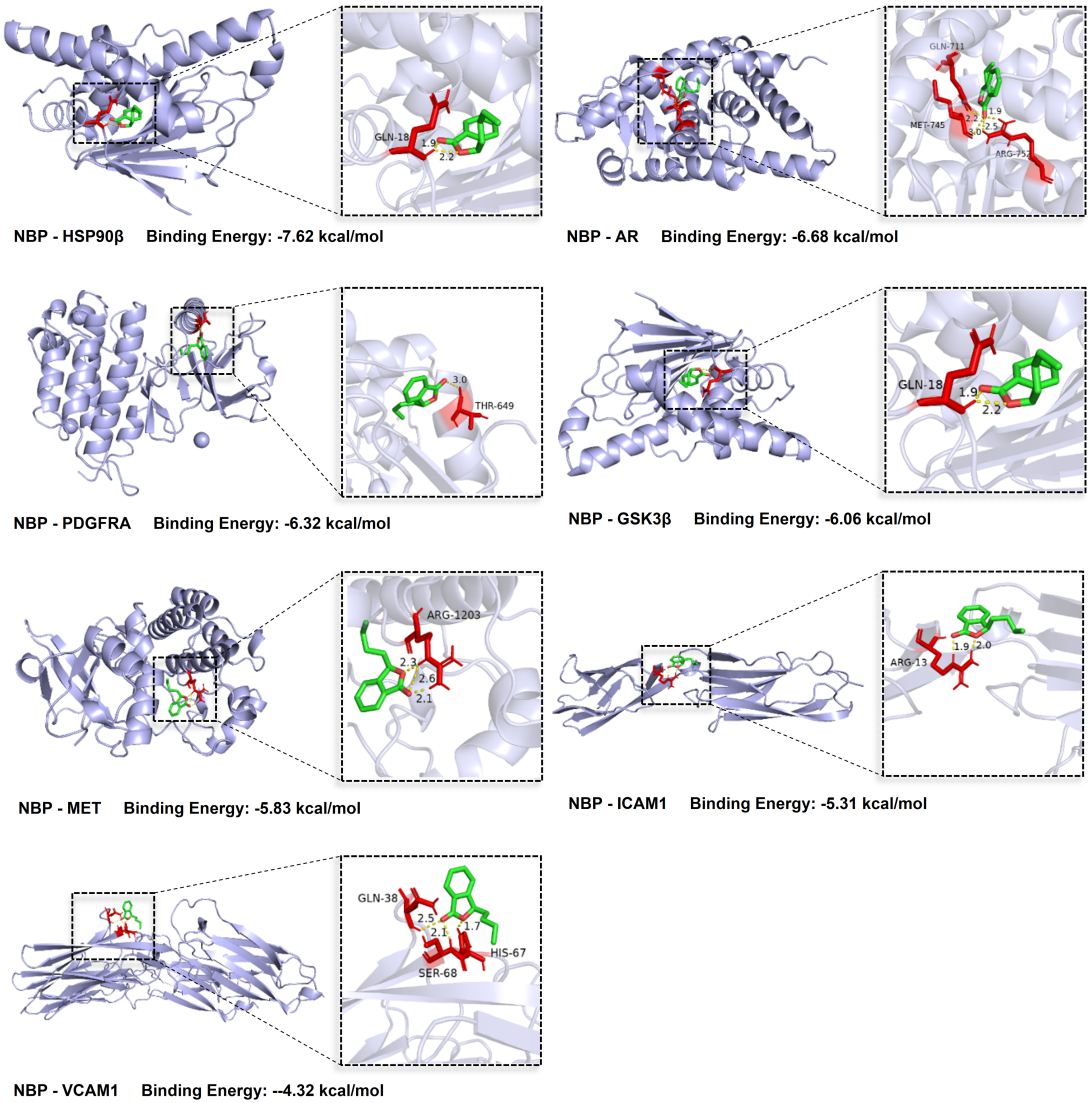
3D binding diagrams and hydrogen bond interactions between NBP and core targets. The green structure represents NBP, shown within the binding pockets of HSP90*β*, PDGFRA, AR, GSK3β, MET, and ICAM1. Yellow lines indicate hydrogen bonds between NBP and the protein targets.

To further validate the potential regulatory interactions between NBP and the core targets associated with *H. pylori* infection, molecular docking was used to evaluate the binding modes and affinities of NBP to the seven core targets. Binding energies are shown in Table 6. Binding energy is a key parameter in molecular docking; the lower the value, the tighter the interaction. Typically, binding energies below −7 kcal/mol indicate strong interactions likely to elicit bioactivity or functional effects. Values below −4.25 kcal/mol suggest good binding affinity. As shown in Figure 6, NBP formed two hydrogen bonds with HSP90β (encoded by *HSP90AB1*) at GLN-18, with a binding energy of −7.62 kcal/mol, indicating strong intermolecular interaction. NBP also exhibited binding energies below −4.25 kcal/mol with PDGFRA, AR, GSK3β (encoded by *GSK3B*), MET, ICAM1, and VCAM1, indicating favorable binding activity.

**Table 6:** Binding energies between NBP and core targets.

Protein	AR	GSK3β	HSP90β	ICAM1	MET	PDGFRA	VCAM1
Binding Energy (kcal/mol)	−6.68	−6.06	−7.62	−5.31	−5.83	−6.32	−4.32

## 4 Discussion

This study discovered the potent anti-*H. pylori* activity of NBP, and silk fibroin was used as a carrier material to prepare NBP-loaded silk fibroin nanoparticles (NBP-NPs) using an desolvation precipitation method. The morphology, particle size, surface charge, and molecular structure of NBP-NPs were comprehensively characterized by scanning electron microscopy, dynamic light scattering, Fourier-transform infrared spectroscopy, and X-ray diffraction. The resulting nanoparticles exhibited uniform size and good stability, with an encapsulation efficiency of 45.80 ± 1.5% and drug loading of 15.27 ± 0.8%.

The MIC and MBC assays showed that NBP exhibited effective antibacterial and bactericidal activities against various *H. pylori* strains, and the nanoparticles demonstrated superior anti-*H. pylori* performance. Inhibiting kinetics assay indicated that NBP inhibited *H. pylori* growth in a dose-dependent manner, with an antibacterial effect comparable to that of clarithromycin. Moreover, no antagonistic effects were observed when NBP was combined with four different antibiotics; instead, additive or irrelevant effects were observed. Notably, for certain strains, the FICI value of NBP with specific antibiotics reached 0.625, which is close to the threshold for synergy (FICI ≤ 0.5), highlighting the therapeutic potential of NBP in the clinical management of *H. pylori.*

*Helicobacter pylori* uses its flagella for motility and adhesion, enabling it to move from the gastric lumen to the mucus layer, where it secretes adhesins to establish colonization on host epithelial cells (Huang et al., 2016). Under increased gastric acidity, the flagella often increase swimming speed to protect the bacteria, as they derive energy from the proton motive force under such pH conditions, enhancing colonization in the acidic stomach environment (Ansari and Yamaoka, 2020). The filament structure of the flagella is composed of multiple subunit proteins, with flaA located at the distal region and flaB at the proximal basal area (Ansari and Yamaoka, 2017). Genetic studies have shown that mutation in the *flaA* gene leads to complete loss of flagellar biosynthesis, while *flaB* mutants can form flagella but exhibit reduced motility and adhesion (Clyne et al., 2000; Huang et al., 2016). RT-qPCR results demonstrated that NBP significantly inhibited the expression of *flaA* and *flaB* in *H. pylori* strains ATCC 700392 and SS1, with the most notable suppression observed in *flaB* of the SS1 strain, showing a gene silencing rate of 63.51±8.2%, suggesting that NBP can effectively impair *H. pylori* motility and adhesion.

Nickel is an essential cofactor for urease and hydrogenase, playing a crucial role in *H. pylori* survival and infection (Becker and Skaar, 2014; Camilo et al., 2017). Urease is considered the most important colonization virulence factor of *H. pylori*, and 24 nickel ions are required to activate one functional urease enzyme molecule (Ha et al., 2001). The acquisition of nickel from the host environment involves complex biological processes: nickel ions are first absorbed by outer membrane transport proteins and then transferred into the cytoplasm via the inner membrane transporter nixA (Ernst et al., 2006; Fulkerson and Mobley, 2000; Wolfram and Bauerfeind, 2002). The urease gene cluster includes catalytic units (*ureA/B*), an acid-gated urea channel (*ureI*), and accessory assembly proteins (*ureE–H*), which together mediate urea hydrolysis to neutralize gastric acid (Marcus et al., 2018; Reshetnyak et al., 2021). When gastric pH drops, the ureI channel opens, allowing urease to convert urea into CO₂, NH₃, and carbamate, which further hydrolyzes into NH₃ and carbonate. The released NH₃ neutralizes gastric acid, promoting *H. pylori* colonization and growth (Huang et al., 2016). Therefore, inhibiting urease activity is a key strategy for eradicating *H. pylori*. Urease activity assays showed that NBP dose-dependently reduced urease activity in both ATCC 700392 and SS1 strains. PCR analysis confirmed the downregulation of *ureA, ureB, ureE–H*, and *nixA* at the molecular level after drug treatment, with *nixA* in SS1 being particularly affected—showing a 62.40% inhibition rate by the nanoparticles. Notably, the nanoparticles showed a smaller absorbance-time change rate and stronger gene silencing effects, confirming the advantages of nanotechnology in enhancing the suppression of *H. pylori* virulence factors.

The cytotoxin-associated gene product CagA is one of the most extensively studied virulence factors of *H. pylori*, located within the cag pathogenicity island (cagPAI) (Noto and Peek, 2012), which encodes not only CagA but also the type IV secretion system (T4SS) (Odenbreit et al., 2000). Among the seven secretion systems in *H. pylori*, types III and IV have transmembrane delivery capabilities, allowing direct injection of bacterial molecules into host cytoplasm. The T4SS, using a pilus-based structure, can directly deliver DNA or CagA protein into host cells, promoting malignant transformation (Baj et al., 2020; Rohde et al., 2003). After entering host cells, CagA binds to SH2-domain-containing proteins via its C-terminal region, inducing tyrosine phosphorylation and leading to dysregulation of the Extracellular Signal-Regulated Kinase (ERK) signaling pathway, Src Family Kinases (SFK) inhibition, and release of the proinflammatory cytokine IL-8 (Bridge et al., 2017; Higashi et al., 2002; Tsutsumi et al., 2003). CagA also promotes gastric ferroptosis through ether lipid biosynthesis and enhances immune escape by upregulating PD-L1 in exosomes, contributing to the aggressiveness and poor prognosis of gastric cancer (Peng et al., 2024; Wang et al., 2023).

Another major virulence factor, Vacuolating cytotoxins A (VacA), disrupts host cell membranes by forming vacuoles, significantly enhancing urease penetration and impairing T-cell activity in the lamina propria, weakening host immune responses (Gebert et al., 2003). RT-qPCR analysis showed that drug treatment significantly reduced the transcription levels of *cagA* and *vacA*, explaining the observed reduction in virulence from a genetic regulation perspective. Western blot results demonstrated that MIC concentrations of the drug inhibited CagA protein expression by 84.51%. Likewise, the nanoparticles exhibited a stronger ability to reduce protein expression (56.50%).

Enrichment analysis of metabolic pathways revealed that the Glycerophospholipid metabolism pathway was significantly activated in the nanoparticle-treated group, suggesting that drug-induced lipid remodeling may be key to membrane damage and bacterial death in SS1 strains. Recent studies show that incorporating various fatty acids into membrane phospholipids affects membrane stability and fluidity. Glycerophospholipids containing monounsaturated, polyunsaturated, or very long-chain fatty acids enhance membrane fluidity (Ma et al., 2004; Stulnig and Zeyda, 2004). Ong et al. demonstrated that the acyl chains of phosphatidylethanolamine (PE) can inhibit bacterial adhesion by remodeling membrane lipids, potentially eradicating *H. pylori* (Ong et al., 2024). Additionally, omega-3 and omega-6 fatty acids exhibit strong anti-inflammatory effects in *H. pylori*-infected gastric epithelial cells, possibly via membrane-associated signaling proteins (Lee et al., 2014). Volcano plot analysis showed that many sn-glycero-3-PE derivatives and unsaturated fatty acids (e.g., cis-7-Hexadecenoic acid, Heptadecenoic acid, 10-Undecenoic acid) were upregulated following drug treatment, suggesting that NBP may damage *H. pylori* membranes by activating Glycerophospholipid metabolism and Biosynthesis of unsaturated fatty acids.

According to the acid adaptation hypothesis (Xia and Palidwor, 2005), *H. pylori*’s acid resistance may involve increased use of positively charged lysine in membrane proteins to block proton influx. Metabolite content analysis showed significant reductions in L-Lysine and derivatives of 4-Trimethylammoniobutanoate post-treatment, implying that disruption of Lysine degradation may disturb intracellular pH homeostasis and acid adaptation. Overall, five metabolic pathways discovered in this study may be key to NBP’s inhibitory or lethal effects on *H. pylori*.

The results of network pharmacology analysis and molecular docking indicated that GSK3*β* plays the most significant regulatory role in the protein–protein interaction (PPI) network, followed by HSP90β. Notably, HSP90β exhibited the most stable binding conformation with the active compound NBP, with a binding energy of −7.62 kcal/mol. PDGFRA, AR, and GSK3β also demonstrated favorable binding activity, with increasing binding energies in that order. Therefore, this study primarily focuses on the potential molecular mechanisms by which GSK3β and HSP90β mediate NBP’s anti-*H. pylori* effects.

Abnormal E-cadherin/catenin signaling has been shown to be closely associated with gastric cancer development (Chen et al., 2005; Nabais et al., 2003). Recent studies suggest that *H. pylori* infection inhibits GSK3β activity, which in turn disrupts β-catenin phosphorylation at Ser/Thr residues and its ubiquitin-mediated degradation, leading to its cytoplasmic accumulation and nuclear translocation. This activates β-catenin-dependent LEF/TCF transcriptional activity and drives the abnormal overexpression of Cyclin D1 (Nakayama et al., 2009; Sokolova et al., 2008). Additionally, Zhang et al. found that NBP significantly reduces the levels of phosphorylated-GSK3β/total GSK3β and β-catenin (by 12.12%) in the mouse hippocampus (Zhang et al., 2016). These findings suggest that NBP may counteract *H. pylori*-induced β-catenin signaling dysregulation by restoring GSK3β activity, providing a theoretical basis for further research into its role in modulating bacterial infection.

Nicotinamide adenosine dinucleotide phosphate (NADPH) oxidase and the mitochondrial electron transport chain are major sources of reactive oxygen species (ROS). The former catalyzes the reduction of O₂ into O₂^−^· or H₂O₂ via transmembrane electron transfer, thus promoting oxidative stress and inflammatory responses (Vermot et al., 2021). Further studies have shown that *H. pylori* can induce the translocation of HSP90β from the cytosol to the membrane in gastric epithelial AGS cells, thereby activating the small G protein Rac1 (a component of the NADPH oxidase complex) (Cha et al., 2010). Although the regulatory mechanism of NBP on HSP90β has not yet been reported, molecular docking analysis suggests a stable, high-affinity binding conformation between the two. This implies that NBP may modulate the NADPH oxidase signaling pathway via targeting HSP90β, thereby interfering with *H. pylori*-induced gastric mucosal damage.

## 5 Conclusion

In summary, this study systematically elucidates the potential mechanisms by which NBP exerts anti-*H. pylori* effects. NBP significantly weakens the pathogen’s colonization and virulence by disrupting bacterial structure, inhibiting urease activity, and downregulating the expression of key virulence and motility-related genes. Through untargeted metabolomics combined with KEGG pathway enrichment analysis, five core metabolic pathways significantly affected by NBP intervention were identifiedNetwork pharmacology discovered seven core targets, all of which showed stable binding conformations with NBP.

In addition, the desolvation method used in this study produced nanoparticles with uniform size and good stability, exhibiting enhanced antibacterial and bactericidal activity. These findings not only provide a molecular basis for the anti-*H. pylori* effects of NBP but also offer valuable insights for future *in vivo* pharmacodynamic evaluations and host-targeted therapeutic strategies.

## Data Availability

Existing datasets are available in a publicly accessible repository: https://doi.org/10.6084/m9.figshare.31034203.

## Footnotes

1
https://www.metaboanalyst.ca/

2
https://pubchem.ncbi.nlm.nih.gov/

3
http://www.swisstargetprediction.ch/

4 https://www.omim.org/

5
https://www.disgenet.org/

6
https://www.genecards.org/

7
https://bioinfogp.cnb.csic.es/tools/venny/

8
https://string-db.org/

9 https://metascape.org/

10
https://www.bioinformatics.com.cn/

11
https://www.rcsb.org/

## Glossary

AA: Acetohydroxamic acid
AMO: Amoxicillin
ATCC: American Type Culture Collection
BHI: Brain heart infusion
CFU: Colony-forming units
CLR: Clarithromycin; FBS, fetal bovine serum
FICI: Fractional inhibitory concentration index
FT-IR: Fourier transform infrared spectroscopy
HPLC: High-performance liquid chromatography
***H. pylori***: *Helicobacter pylori*
LEF: Levofloxacin
McF: McFarland
MBC: Minimum bactericidal concentration
MIC: Minimum inhibitory concentration
MTZ: Metronidazole
NBP: DL-3-n-Butylphthalide
OD: Optical density
PBS: Phosphate-buffered saline
RT-qPCR: Reverse transcription quantitative polymerase chain reaction
SEM: Scanning electron microscope
SF: Silk fibroin
XRD: X-ray diffraction
